# Enhanced Performance of Near-Infrared Perovskite Light-Emitting Diodes with PEDOT:PSS Buffer Layer

**DOI:** 10.3390/molecules31121984

**Published:** 2026-06-06

**Authors:** Shaowen Chen, Xiaodong Chi, Piaoyang Shen, Chaoyu Xiang

**Affiliations:** 1 School of Materials Science and Chemical Engineering, Ningbo University, Ningbo 315211, China; 2 Laboratory of Optoelectronic Information Technology and Devices, Ningbo Institute of Materials Technology and Engineering, Chinese Academy of Sciences, Ningbo 315201, China; 3 Hangzhou Bay Laboratory of Advanced Nano-Optoelectronic Materials and Devices, Qianwan Institute of CNITECH, Ningbo 315336, China

**Keywords:** near-infrared, buffer layer, perovskite light-emitting diodes, balanced carrier injection

## Abstract

Perovskite light-emitting diodes (PeLEDs) have attracted considerable attention due to their outstanding electroluminescent properties and have achieved remarkable progress. However, charge injection imbalance remains a major obstacle limiting the performance of near-infrared (NIR) PeLEDs. Herein, we propose inserting a poly(3,4-ethylenedioxythiophene)-poly(styrenesulfonate) (PEDOT:PSS) buffer layer between ITO and Zinc oxide (ZnO) to reduce electron injection. This layer also acts as a substrate to modulate ZnO surface roughness, thereby improving perovskite film quality. Through this optimization, the device’s external quantum efficiency (EQE) increases from 20% to 22%, and its T_50_ operational lifetime extends from 3.4 h to 17.8 h. Importantly, we successfully integrate the PEDOT:PSS buffer layer into scalable fabrication, demonstrating NIR-PeLEDs with a uniform emission area of 2500 mm^2^.

## 1. Introduction

Metal halide perovskites have emerged as promising candidates for next-generation light-emitting diodes due to their excellent optoelectronic properties [1,2,3,4,5], such as high photoluminescence quantum yield (PLQY) and tunable emission wavelength with narrow full width at half maximum [6]. In the past decade, the external quantum efficiency (EQE) of near-infrared perovskite light-emitting diodes (NIR-PeLEDs) has been rapidly improved through methods such as optimization of charge transport layers [7,8], perovskite defect passivation [9], device structure optimization [10,11], and design of light coupling structures [12]. The record EQE of NIR-PeLEDs has exceeded 30% [13]. Despite the rapid improvement in peak efficiency, the commercial application of NIR-PeLEDs remains hindered by challenges in scaling up, which is intrinsically linked to charge injection imbalance [14,15].

Zinc oxide (ZnO) is widely used as an electron transport layer (ETL) in NIR-PeLEDs due to its high optical transparency, electron mobility, and processability [16]. However, the hole mobility of typical HTLs (e.g., poly[(9,9-dioctylfluorenyl-2,7-diyl)-alt-(4,4′-(N-(4-butylphenyl)diphenylamine))](TFB) is far lower than ZnO’s electron mobility [17], causing carrier imbalance and electron accumulation in the perovskite emissive layer. This drives non-radiative recombination and degrades device performance [18]. To mitigate this issue, researchers attempted to reduce the electron injection in the perovskite layer through interface engineering strategies [19,20,21]. For example, Peng et al. inserted PMMA between ZnO and perovskite as a charge blocking layer (CBL) to restrict electron transfer and achieve balanced charge injection. The fabricated device had an EQE of up to 20.5% [22]; Li et al. improved the performance of PLED by atomically layering and depositing ultra-thin alumina (Al_2_O_3_) CBL between ZnO and perovskite layers. Under the optimal Al_2_O_3_ CBL condition, the peak EQE of the NIR-PeLED was 17.0% [23]. However, the traditional interface engineering strategies aimed at reducing electron injection have some limitations. For example, using Al_2_O_3_ as a charge blocking layer usually could alleviate charge imbalance, but unintentionally damaged the surface chemical properties of ZnO and reduced the deprotonation ability of ZnO [24], resulting in poor perovskite film quality; using PMMA as a charge blocking layer is limited by the preparation process and struggles to achieve scalable fabrication. Moreover, in lead-based perovskites, the interface contact between the transport layer and the perovskite affects crystal nucleation, growth, carrier transport, and device stability. Traditional interface treatment methods tend to introduce interface defects, residual stress, and poor uniformity, which hinders large-scale production [25]. Given these limitations, an alternative and underexplored approach is to modulate the perovskite crystallization by engineering the growth substrate of the ZnO film, rather than directly modifying the ZnO/perovskite interface. Additionally, most NIR-LED research focuses on small-area devices prepared by spin-coating, with limited progress in scalable fabrication [17,26,27,28]. At present, the research on large-area NIR-PeLEDs mainly focuses on the spin-coating method and inkjet printing. For example, Chen et al. used materials with shallower ionization potential as the hole transport layer to improve the charge balance and efficiency of the device, and fabricated large-area (900 mm^2^) NIR-PeLEDs with an EQE of 12.1% by spin coating method [27]; Xiao et al. suppressed the coffee ring effect by adjusting the fluid and evaporation dynamics of the perovskite wet layer, and fabricated a large-area PeLED (4 × 7 cm^2^) with an EQE of 14.3% through inkjet printing [29]. The blade coating method, with its high material utilization rate, is an ideal path for PeLEDs to move towards low-cost and large-scale industrialization. However, due to the limitations of the precision of existing fabrication processes and film quality, achieving spatial uniformity of the perovskite layer is difficult, easily forming local defects, thickness variations, and consequent leakage current paths [30].

In this work, we innovatively proposed adding PEDOT: PSS between ITO and ZnO as a buffer layer to reduce electron injection. Since PEDOT: PSS is located between ITO and ZnO, compared to traditional interface treatment, it avoids the influence on the deprotonization ability of ZnO. Our strategy of using PEDOT:PSS as the buffer layer enables scalable manufacturing. Moreover, by adjusting the ZnO film, the quality of the perovskite film was improved. This synergistic effect significantly enhances the crystallinity and uniformity of the perovskite emissive layer, leading to suppressed non-radiative recombination and improved charge balance. Through this optimization, the device’s EQE increases from 20% to 22%, and the T_50_ lifetime improves from 3.4 h to 17.8 h. Furthermore, capitalizing on the buffer layer’s exceptional ability to suppress leakage currents, we successfully scale up this architecture to fabricate large-area NIR-PeLEDs. The final device demonstrated a uniform emission area of 2500 mm^2^, verifying the feasibility of the buffer layer strategy in the scalable fabrication of NIR-PeLEDs. This result provides a new strategy for the practical application of PeLEDs in large-area display and lighting technologies.

## 2. Results and Discussion

**Figure 1:**
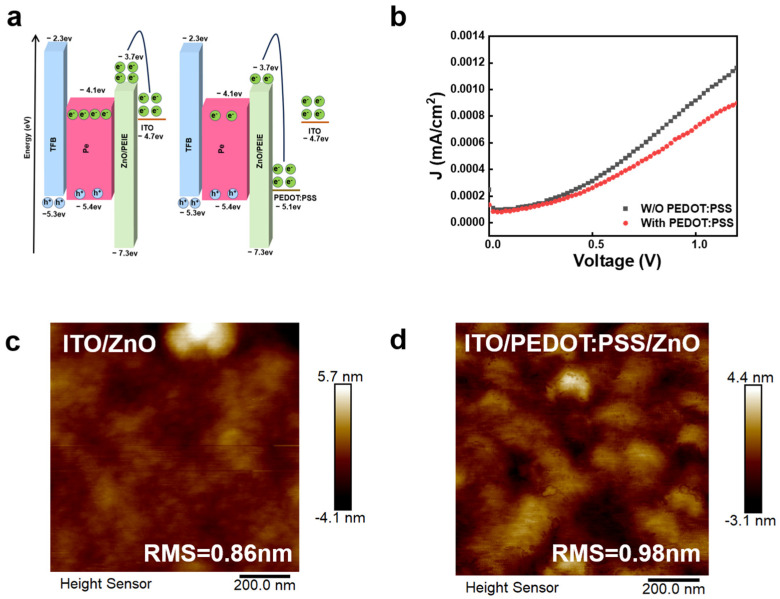
(**a**) Schematic illustration of the PEDOT:PSS layer buffer excess electrons (energy level values are taken from reference [31]); (**b**) Current density-voltage characteristics of the electron-only device; (**c**) AFM image of the ZnO film on ITO substrate; (**d**) AFM image of the ZnO film on PEDOT:PSS substrate.

Traditional NIR-PeLEDs typically utilize ZnO as the ETL and organic materials like TFB as the HTL. However, the marked difference in carrier mobility between these transport layers results in severe carrier injection imbalance. Carrier injection imbalance represents one of the most critical factors limiting both the performance and operational stability of NIR-PeLEDs. This carrier injection imbalance leads to an excessive accumulation of electrons within the perovskite emissive layer, particularly under high-current-density operation. Electron accumulation triggers a series of detrimental effects: the accumulated electrons fail to undergo timely radiative recombination with holes, instead undergoing non-radiative recombination at defect sites, which results in significant efficiency loss. To address this, we add a PEDOT:PSS buffer layer with a large barrier between ITO and ZnO to reduce electron injection and reduce the accumulation of carriers in the light-emitting layer (as shown in Figure 1a). To verify that the PEDOT:PSS buffer layer can effectively reduce electron injection, we fabricate electron-only devices with the structure of ITO/ZnO/PEIE/Pe/TPBi/LiF/Al. As shown in Figure 1b, the electron-only devices containing the PEDOT:PSS buffer layer exhibit lower current density at the same voltage, which directly confirms that the PEDOT:PSS buffer layer can effectively reduce electron injection. Through this regulation of electron injection, the carrier injection imbalance is alleviated.

To investigate whether PEDOT:PSS affects the chemical interaction between ZnO/PEIE and the perovskite precursor, we have conducted Fourier-transform infrared (FTIR) spectroscopy measurements. The relative peak intensity of C-N is 1666 cm^−1^, serving as an indicator for monitoring the deprotonation process of FAI molecules. As shown in Figure S3, we compared the FTIR spectra of the perovskite films deposited on ZnO/PEIE and ZnO/PEIE/PEDOT:PSS after 3 min and 10 min of annealing. The analysis revealed that without the PEDOT:PSS buffer layer, the C-N peak intensity dropped to 27.9% of that at 3 min after 10 min of annealing. In contrast, with the addition of the PEDOT:PSS buffer layer, the C-N peak intensity dropped to 26.5% of that at 3 min. The slight difference in the decay rate indicates that the presence of PEDOT:PSS does not hinder the deprotonation ability of the ZnO/PEIE layer. This finding highlights the unique advantages of using the PEDOT:PSS buffer layer. To further investigate the impact of introducing the PEDOT:PSS buffer layer on the upper films, we perform morphological and crystallographic characterizations on the fabricated films. Figure 1c and Figure 1d present atomic force microscopy (AFM) images of ZnO films deposited on ITO and PEDOT:PSS substrates, respectively. As illustrated in Figure 1c, the ZnO film grown directly on the bare ITO substrate exhibits a relatively flat and smooth surface topography. While a smooth surface is typically desirable, it proves detrimental in the context of perovskite crystallization. When perovskite films are deposited onto such a flat ZnO surface, lattice mismatch often induces significant tensile strain within the perovskite crystal lattice. This strain weakens chemical bonding interactions, promotes the formation of chemically unstable structures, and, more critically, lowers both the defect formation energy and the activation energy for ion migration [32]. Consequently, this facilitates the generation of non-radiative recombination centers and accelerates device degradation, thereby compromising long-term operational stability. In stark contrast, the ZnO film deposited on the PEDOT:PSS substrate displays a distinctly rougher surface morphology with an increased root-mean-square (RMS) roughness (Figure 1d). After adding PEDOT:PSS buffer layer, the surface RMS of ZnO increases from 0.86 nm to 0.98 nm. To verify the statistical significance of the observed roughness variation, comprehensive AFM measurements were performed across multiple regions of each sample. As illustrated in Figure S5, the ITO/ZnO sample exhibits relatively smooth surfaces with RMS values consistently around 0.8 nm. The ITO/PEDOT:PSS/ZnO sample exhibited a rougher surface morphology. The RMS value of the sample was significantly higher, ranging from 0.98 nm to 1.08 nm. Furthermore, we also measured the contact angles of ZnO on ITO and ZnO on PEDOT: PSS. As shown in Video S1 and Figure S7, ZnO presents a fully spread-out state on ITO, and the contact angle cannot be measured. However, a small contact angle can be measured on PEDOT:PSS. Compared to ITO, ZnO has lower wettability on the PEDOT:PSS surface, which leads to an increase in the surface roughness of ZnO. The higher surface roughness provides more nucleation sites for the perovskite, thereby forming a dense and smaller-grained perovskite film.

The improvement of surface roughness can alleviate harmful tensile strains. The uneven surface creates an uneven stress distribution, with stress concentrating at the protrusions and being effectively released in the grooves. This unique topography acts as a mechanical buffer, thereby alleviating the overall average residual stress on the perovskite film and inhibiting the formation of strain-induced defects. The XRD peak position of the (111) crystal plane of α-phase FAPbI_3_ is located at 14°. Through the analysis of the XRD peak shift (Figure S6), it was found that the (111) crystal plane of the perovskite shifted from the original 13.63° to 13.71°, which is closer to 14°. This indicates that the strain of the perovskite decreases after the addition of the PEDOT:PSS buffer layer.

**Figure 2:**
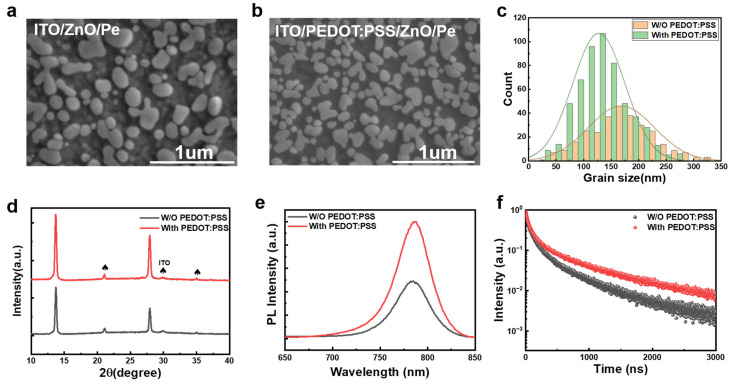
(**a**) SEM image of the perovskite film without the PEDOT:PSS buffer layer; (**b**) SEM image of the perovskite film with the PEDOT:PSS buffer layer; (**c**) Grain size distribution of perovskite crystallites; (**d**) XRD pattern; (**e**) PL spectrum of the perovskite film; (**f**) TRPL decay of the perovskite film.

Subsequently, we analyze the effects of the PEDOT:PSS buffer layer on the grain size and distribution of perovskite films using scanning electron microscopy (SEM). As shown in Figure 2a,b, the addition of the PEDOT:PSS buffer layer leads to significant changes in the grain size and film coverage, with improved film coverage. The introduction of the PEDOT:PSS buffer layer alters the grain growth during film formation. In contrast to the uncontrolled rapid crystallization observed in the absence of the buffer layer, the introduction of the PEDOT:PSS buffer layer provides a more favorable nucleation environment for the perovskite. This modulation promotes the formation of a denser array of smaller, yet highly uniform, crystallites. Grain size statistics (Figure 2c) reveal that the average grain size decreases from 170 nm to 140 nm, resulting in stronger spatial confinement and more favorable conditions for radiative recombination [33].

Subsequently, to gain deeper insights into the crystallographic evolution induced by the PEDOT:PSS buffer layer, we evaluate the effect of the PEDOT:PSS buffer layer on the crystallinity of the perovskite films using X-ray diffraction (XRD) analysis. As illustrated in Figure 2d, the perovskite film without the PEDOT:PSS buffer layer exhibits characteristic diffraction peaks near 14° and 28°, which correspond to the (111) and (222) crystal planes of the photoactive α-phase FAPbI_3_ [12], respectively. Notably, the incorporation of the PEDOT:PSS buffer layer results in an enhancement in the intensity of (111) and (222) diffraction peaks, while the pattern remains free of any impurity or non-perovskite phase signals. This significant intensification suggests that the buffer layer not only promotes the growth of highly crystalline domains but also induces a preferential orientation along the (111) direction. Such improved crystallinity and phase purity are indicative of a reduced density of grain boundary defects and trap states, which are essential for minimizing non-radiative recombination losses and enhancing charge transport within the device.

To comprehensively evaluate the impact of the PEDOT:PSS buffer layer on the optoelectronic quality of the perovskite films, we conduct a series of optical characterizations. As shown in Figure S1 and Figure 2e, both the UV-vis absorption and steady-state photoluminescence (PL) spectra exhibit the characteristic features of high-quality three-dimensional α-FAPbI_3_, with a distinct PL peak centered at 790 nm. This confirms that the introduction of the buffer layer does not alter the intrinsic bandgap of the perovskite material. Furthermore, time-resolved photoluminescence (TRPL) measurements are performed to gain deeper insights into the carrier recombination dynamics (Figure 2f). The TRPL decay was fitted using a bi-exponential model. The detailed fitting parameters (τ_1_, τ_2_, A1, A2) and the calculated average lifetime (τ_ave_) for the TRPL curves are summarized in Table S2. As shown in the table, the average lifetime of the perovskite film increases significantly from 217.04 ns (without PEDOT:PSS) to 353.51 ns (with PEDOT:PSS). This enhancement in PL lifetime suggests a substantial reduction in the trap state density and effective suppression of non-radiative recombination centers.

**Figure 3:**
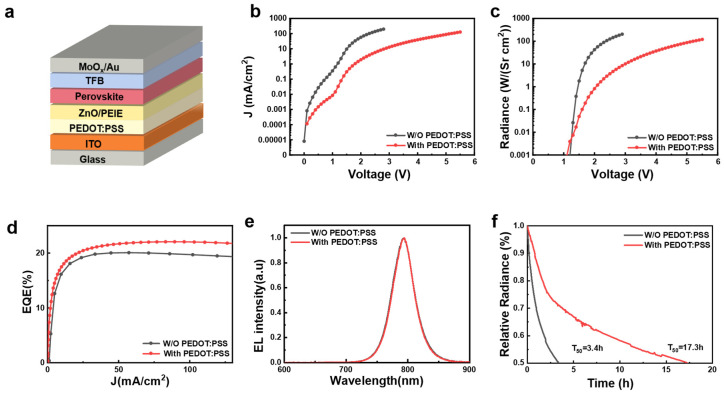
(**a**) Schematic illustration of the PeLED structure; (**b**) Current density-voltage (J-V) curves; (**c**) Radiance-voltage (R-V) curves; (**d**) EQE as a function of current density; (**e**) EL spectra; (**f**) Device stability measured under a constant current density of 100 mA cm^−2^.

To systematically verify the efficacy of the PEDOT:PSS buffer layer in optimizing device performance, we fabricate NIR-PeLEDs with a configuration of ITO/PEDOT:PSS/ZnO-PEIE/Pe/TFB/MoO_3_/Au (schematic illustrated in Figure 3a). We use a Cs-doped FAPbI_3_ perovskite with the composition Cs_0.05_FA_0.95_PbI_3_, modified with 5-aminovaleric acid (5AVA) as an additive. A comprehensive comparative study is conducted between devices with and without the buffer layer to elucidate the feasibility of this buffer layer strategy.

The thickness of PEDOT:PSS is optimized by adjusting the rotational speed of PEDOT:PSS (3000–6000 rpm) (Figure S4). When the rotational speed reaches 5000 rpm, the device efficiency is at its peak, reaching 22%. The comparison of the devices’ electrical performance parameters is shown in Figure 3b–d. The J-V curves reveal that the leakage current is significantly reduced after incorporating PEDOT:PSS as a buffer layer. This reduction in leakage indicates reduction in defect density and the blocking of shunt pathways, which are often detrimental to device performance. Consequently, reduction in leakage current translates directly into superior electroluminescent metrics. While the device without buffer layer demonstrates a commendable peak external quantum efficiency (EQE) of 20.0% and a maximum radiance of 196.8 W sr^−1^ m^−2^, the device with the PEDOT:PSS buffer layer demonstrates markedly enhanced performance. Specifically, the peak EQE increases to 22.0%, and the maximum radiance increases to 226.9 W sr^−1^ m^−2^, underscoring the pivotal role of the buffer layer in improving device performance. As shown in Figure 3e, the electroluminescence (EL) spectra of both devices nearly overlap completely, with identical main emission peaks at 795 nm and nearly the same FWHM. Furthermore, we evaluate the operational stability of the devices under constant current driving. As illustrated in Figure 3f, under a constant current density of 100 mA cm^−2^, the device incorporating the PEDOT:PSS buffer layer displays superior stability, achieving a T_50_ lifetime of 17.3 h, approximately five times longer than that of the device without the PEDOT:PSS buffer layer (3.4 h). This enhancement in both efficiency and stability is primarily attributed to the effective regulation of electron injection by the PEDOT:PSS layer. By acting as an electron injection buffer, the layer balances the carrier and prevents excessive electron accumulation at the interface. This balance not only maximizes radiative recombination but also mitigates device degradation caused by non-radiative recombination, thereby significantly extending the device’s operational lifetime. As previously discussed, the PEDOT:PSS layer functions as a critical electron injection buffer, effectively moderating the electron to prevent excessive injection. The introduction of the PEDOT:PSS buffer layer establishes a more balanced carrier injection which is pivotal for high-performance emission. By balancing the electron and hole, the strategy not only minimizes the probability of non-radiative recombination, but also alleviates the accumulation of excessive carriers within the emissive layer. This reduction in accumulation electronics significantly alleviates the non-radiative feature of the device, thereby simultaneously boosting both the peak external quantum efficiency and the long-term operational stability. To verify the repeatability of the devices, we conducted additional statistical tests on the results (under the same conditions). As shown in Figure S2, the EQE value distribution of the devices with the PEDOT:PSS buffer layer was higher than that of the devices without the PEDOT:PSS buffer layer. The average EQE increased from 19.0% to 21.4%. This indicates that the improvement in device performance is relatively stable and is not affected by common experimental variations, such as measurement errors. A comprehensive comparison of recent NIR-PeLEDs is provided in Table S1, detailing key parameters such as device architecture, emission wavelength, EQE, operational lifetime (T_50_), active area, and interface engineering strategies. As can be seen in the table, in the interface processing strategy, the addition of the PEDOT:PSS buffer layer demonstrates great potential for achieving highly efficient NIR-PeLEDs.

**Figure 4:**
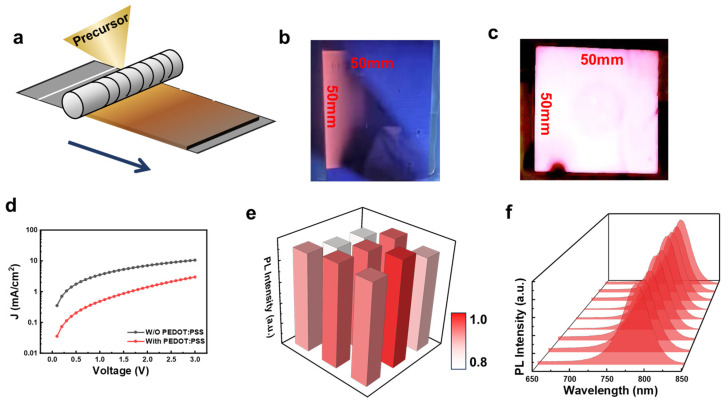
(**a**) Schematic illustration of the blade-coating method; (**b**) Operational photograph of the 2500 mm^2^ device with PEDOT:PSS buffer layer; (**c**) Operational photograph of the 2500 mm^2^ device with PEDOT:PSS buffer layer; (**d**) J-V curves of the 2500 mm^2^ device; (**e**) Fluorescence intensity distribution at nine positions across the perovskite film; (**f**) Fluorescence peak positions at nine positions across the perovskite film.

Leakage current has long been a critical bottleneck impeding the reliable operation and commercial viability of large-area PeLEDs. In order to verify the feasibility of the PEDOT:PSS buffer layer strategy in the scalable fabrication of NIR-PeLEDs, we transition from laboratory-scale spin-coating to a more industrially relevant blade-coating process (Figure 4a). This technique, while promising for scalable manufacturing, often exacerbates film inhomogeneity and defect formation. By integrating the PEDOT:PSS buffer layer into this blade-coating fabrication workflow, we successfully overcome leakage current, demonstrating the strategy’s potential to enable high-performance, large-area optoelectronic devices. As illustrated in Figure 4b,c, under forward bias, a striking contrast in emission area is observed between the two device configurations. The device without the PEDOT:PSS buffer layer exhibits highly non-uniform, localized emission with distinct dark spots. This inhomogeneity is primarily attributed to the inherent limitations of scalable fabrication processes, where achieving uniform film coverage is challenging. In sharp contrast, the device incorporating the PEDOT:PSS buffer layer displays uniform, bright luminescence across the entire active area. As quantitatively illustrated in the current density-voltage (J-V) curves (Figure 4d), the leakage current in the device with the PEDOT:PSS buffer layer is significantly suppressed compared to the device without the PEDOT:PSS buffer layer. This suggests that the buffer layer effectively reduces defect density and blocks the formation of shunt pathways, thereby ensuring uniform device performance even at larger scales. To quantitatively evaluate device uniformity, we select nine uniformly distributed positions on the perovskite film with the PEDOT:PSS buffer layer and measure the PL intensity (Figure 4e) and fluorescence peak position (Figure 4f) at these locations. The PL intensity and fluorescence peak position show minimal fluctuations across all measured positions. This result demonstrates the advantages of incorporating the PEDOT:PSS buffer layer in fabricating high-quality and large-scale uniform perovskite films and devices. This work is expected to promote the application of PeLEDs in display and lighting fields.

## 3. Materials and Methods

Materials: FAI (99.99%, Sigma-Aldrich, Saint Louis, MO, USA), PbI2 (99.999%, Macklin, Shanghai, China), CsI (99.99%, Sigma-Aldrich), PEIE (Sigma-Aldrich, 37 wt%), 1H,1H-perfluorohexylamine (PFHA (Frankfort, KY, USA), 97%), TFB (ADS, molecular weight 85,000), MoO_3_ (99.999%, Macklin), Au (99.999%, Alfa Aesar, Ward Hill, MA, USA), dimethylformamide (J&K Scientific, Beijing, China), FMSA (Alfa Aesar), 5AVA (97%, Sigma-Aldrich), isopropanol (99.9%, J&K Scientific), zinc acetate dihydrate (99.99%, Sigma-Aldrich), ethanolamine (99.5%, Sigma-Aldrich), and 2-methoxyethanol (99.8%, Sigma-Aldrich). ITO glass with specific electrode patterns was purchased commercially. All materials were used directly without any purification.

Device Fabrication: Patterned ITO glass was sequentially ultrasonically cleaned with deionized water, isopropanol, and acetone for 15 min each. After drying, the substrate was treated with UV-O3 for 15 min, followed by deposition of a PEDOT:PSS film by spin-coating at 5000 rpm for 30 s and annealing in air at 150 °C for 10 min. The ZnO precursor was prepared by dissolving ethanolamine (0.5 M) and zinc acetate dihydrate (0.5 M) in 2-methoxyethanol (1 mL), which was then spin-coated onto the ITO substrate at 5500 rpm and annealed at 180 °C for 15 min to form the electron transport layer. The PEIE stock solution (37 wt.%) was diluted to 0.05 wt.% in isopropanol, deposited onto the ZnO film by spin-coating at 5000 rpm for 30 s, and annealed in air at 100 °C for 10 min. Subsequently, perovskite and TFB were spin-coated in a nitrogen-filled glovebox. The perovskite precursor was prepared by dissolving FAI (Formamidinium iodide; the primary A-site cation source): CsI (Cesium iodide; used for phase stabilization): PbI_2_ (Lead iodide; the B-site cation and anion source): 5AVA (5-Aminovaleric acid; a passivating additive) at a molar ratio of 0.05:2.4:1:0.7 in DMF solution (concentration of 0.07 M). The filtered perovskite precursor solution was dropped onto the ZnO-PEIE substrate and spin-coated at 3000 rpm for 30 s, followed by thermal annealing at 100 °C for 16 min to obtain the perovskite film. TFB (12 mg) was dissolved in chlorobenzene (1 mL), spin-coated at 3000 rpm for 30 s, and thermally annealed at 90 °C for 10 min to prepare the TFB hole transport layer. Finally, MoO_3_ and Au layers were deposited by thermal evaporation.

Device Characterization: The perovskite LED devices were tested under ambient conditions after encapsulation with cover glass. Current density-voltage-luminance (J-V-L) curves, EL spectra, and EQE were recorded simultaneously using a Keithley 2400 light source meter and commercial equipment (XPOY-EQE-350-1100, Guangzhou Xipu Optoelectronics Technology Co., Guangzhou, China). Surface morphology of the perovskite coatings was collected using atomic force microscopy (Bruker (Billerica, MA, USA), Dimension ICON). X-ray diffraction patterns were measured using a Bruker D8 Advance diffractometer under Cu Kα radiation. Photoluminescence lifetime measurements were conducted using a compact near-infrared photoluminescence lifetime spectrometer (Hamamatsu (Hamamatsu, Japan), C12132-37) with a 532 nm pulsed laser. Contact angles were measured using a contact angle meter (Data Physics (Riverside, CA, USA); OCA25). Fluorescence spectra of the perovskite films were measured using HORIBA (Kyoto, Japan) FL3-111 (excitation wavelength: 475 nm), and absorption spectra were obtained using a Perkin-Elmer (Waltham, MA, USA) Lambda 950.

## 4. Conclusions

This work addresses the issue of excessive electron injection by inserting a PEDOT:PSS buffer layer between ITO and ZnO. Additionally, the PEDOT:PSS buffer layer serves as a substrate modulating the ZnO film roughness, thereby improving the quality of the perovskite film. Through this optimization, the EQE of device improves from 20% to 22%, and the T_50_ lifetime increases from 3.4 h to 17.8 h. Furthermore, we introduce the PEDOT:PSS buffer layer strategy into scalable fabrication, successfully preparing large-area NIR-PeLEDs with an emission area of 2500 mm^2^, promoting the application of PeLEDs in display and lighting fields.

## Data Availability

The original contributions presented in this study are included in the article/Supplementary Materials. Further inquiries can be directed to the corresponding authors.

## Supplementary Materials

The following supporting information can be downloaded at: https://www.mdpi.com/article/10.3390/molecules31121984/s1. Refs. [13,17,23,29,34,35,36,37,38] are cited in the Supplementary Materials.
