# Supplementary material for: Enhanced Performance of Near-Infrared Perovskite Light-Emitting Diodes with PEDOT:PSS Buffer Layer
Source: Molecules. 2026 Jun 6;31(12):1984. doi: 10.3390/molecules31121984 (PMC13304203; doi:10.3390/molecules31121984)
Supplement: Supplementary file 1 [file molecules-31-01984-s001.zip › Supplementary Information.pdf]

Supplementary Information

Enhanced Performance of Near-Infrared Perovskite Light-Emitting Diodes with PEDOT:PSS Buffer Layer

Shaowen Chen[1,2,3], Xiaodong Chi[2,3], Piaoyang Shen[2,3*], Chaoyu Xiang[2,3*]

1. School of Materials Science and Chemical Engineering, Ningbo University, Ningbo 315211, China

2. Laboratory of Optoelectronic Information Technology and Devices, Ningbo Institute of Materials Technology and Engineering, Chinese Academy of Sciences, Ningbo 315201, China

3. Hangzhou Bay Laboratory of Advanced Nano-Optoelectronic Materials and Devices, Qianwan Institute of CNITECH, Ningbo 315336, China

Email: xiangchaoyu@nimte.ac.cn.

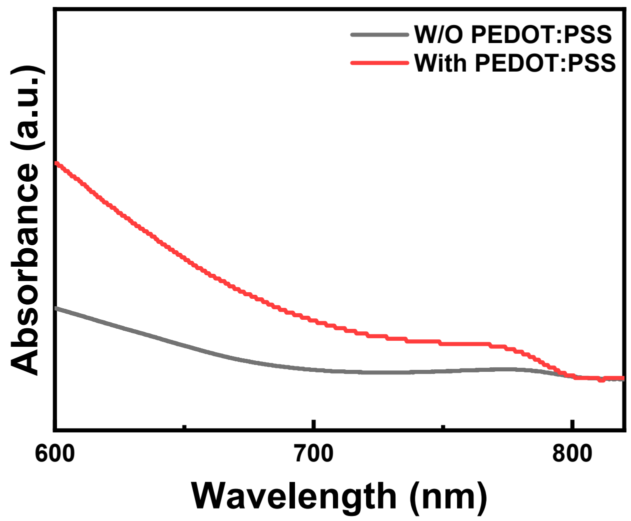

Figure S1. Absorption spectrum of the perovskite films

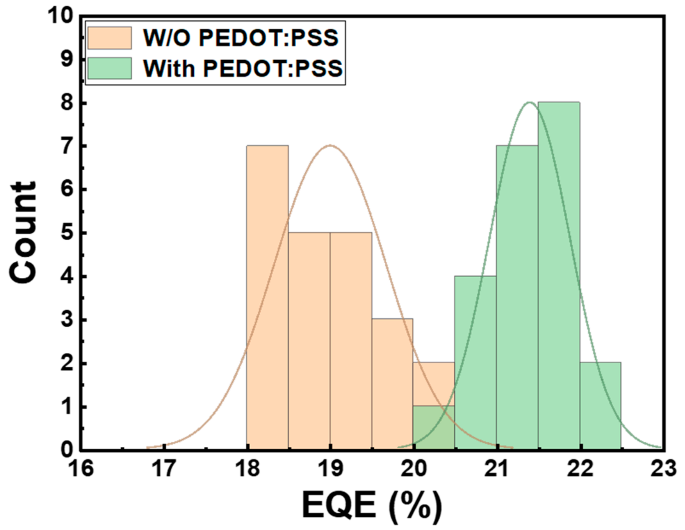

Figure S2. Device performance statistics without PEDOT:PSS buffer layer and with PEDOT:PSS buffer layer

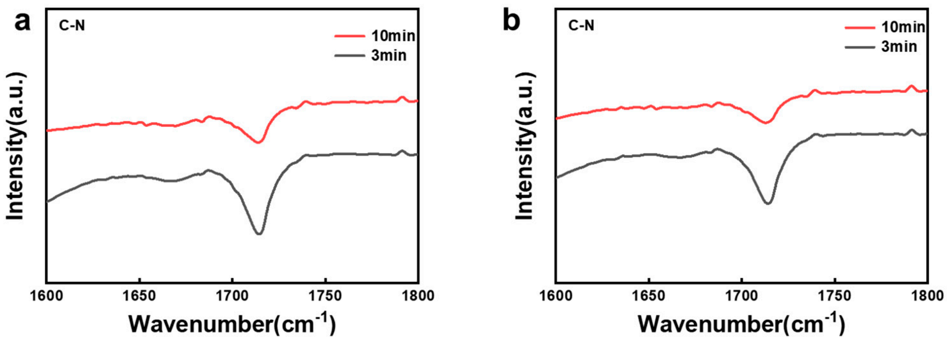

Figure.S3. a) FTIR spectra of perovskite films on ZnO-PEIE (without the PEDOT:PSS) ; b) FTIR spectra of perovskite films on ZnO-PEIE (with the PEDOT:PSS)

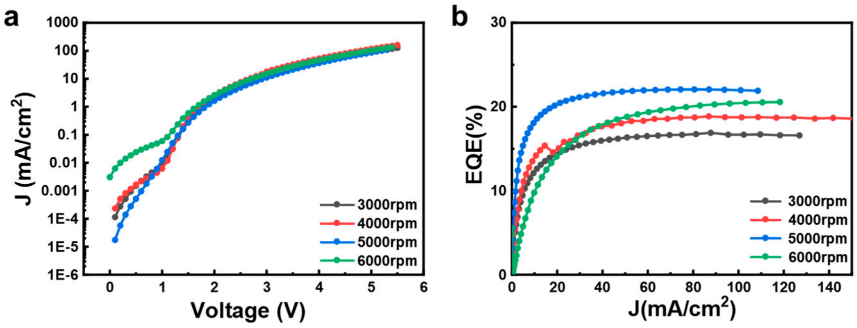

Figure.S4. a) J-V curves of PEDOT at different rotational speeds; b) Current density-EQE curves of PEDOT at different rotational speeds

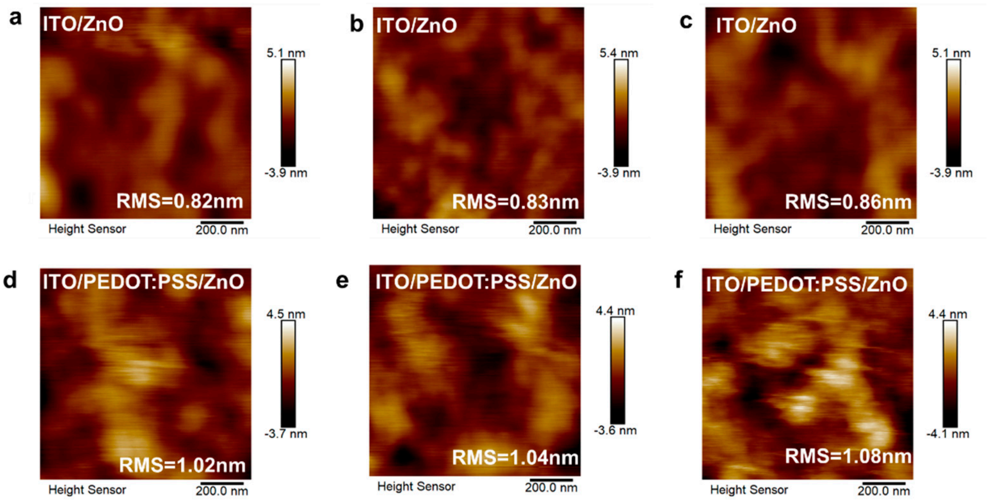

Figure.S5. a-c AFM images for ZnO films with different regions (ITO/ZnO); d-f AFM images for ZnO films with different regions (ITO/PEDOT:PSS/ZnO)

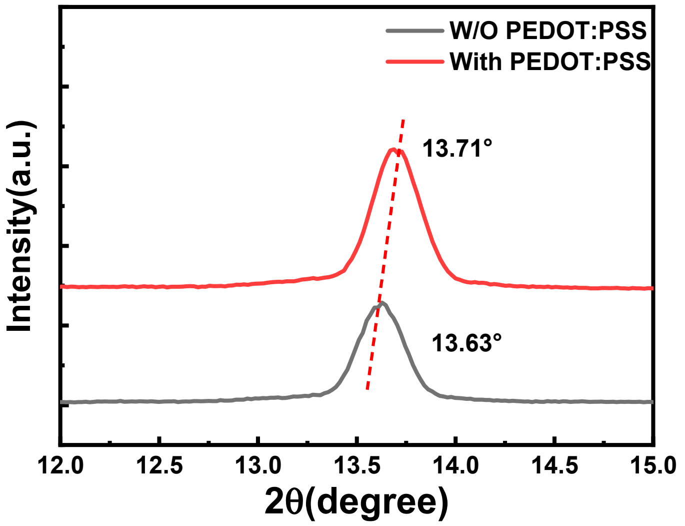

Figure.S6. XRD patterns of perovskite films

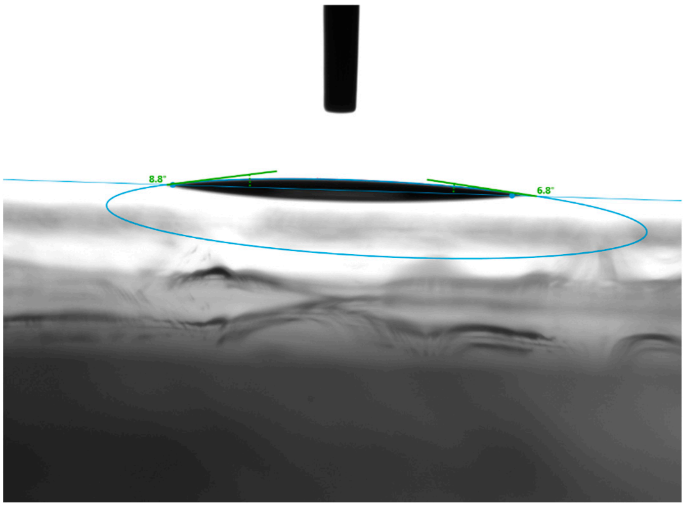

Figure.S7. Contact angle measurement of ZnO on PEDOT:PSS

Table S1 Summary on the representative 3D PeLEDs in NIR region in literature

device structure	interfacial strategy	Peak EQE (%) active area(mm^2)	emission wavelength (nm)	current density (mA cm^{-2}) (Lifetime (h))
Ito/ZnO-PEIE/Pe/TFB/MoOx/Au[1]	(Me-4PACz Surface treatment)	21.98(4)	800	50(T50=47.3)
Ito/ZnO-PEIE/Pe/TFB/MoOx/Au[2]	(AIPZ; Surface treatment)	22.1(4)	806	100(T50=20.3)
Ito/ZnO-PEIE/Pe/TFB/MoOx/Au[3]	(TFA; Surface treatment)	22.32(4)	807	25(T50=103.3)
Ito/ZnO-PEIE/Al2O3/Pe/TFB/MoOx/Au[4]	(Al2O3; Charge blocking layer)	17.0(4)	800	20(T50=43.7)
ITO/ZnO-PEIE/Pe/Poly-TPD/MoO3/Al[5]	No	12.1(900)	799	57 (T80=20)
ITO/PEDOT:PSS/Poly-TPD/PVP/Pe/TPBi/LiF/Al[6]	No	14.3(4)<br>Only display (2800)	769	100(T50=2.59)
ITO/ZnO/PEIE/FASCN/Pe/TFB/MoO3/Al[7]	FASCN buried-interface modification	21.1(4)	797	50(T50=37.5 h)
ITO/Poly-TPD/Pe/ZnMgO)/Ag[8]	No	9.5(4)	850	20(T50=125 h)
Ito/ZnO-PEIE/Pe/TFB/MoOx/Au[9]	No	32.6	801	100(T50=17)

Ito/ZnO-PEIE/Pe/TFB/MoOx/Au(This Work)	(PEDOT:PSS; Buffer layer)	22(4)<br><br>Only display (2500)	795	100(17.8)

1. Wang, W.J.; Liu, W.Z.; Xu, S.H.; Zhou, D.Y.; Peng, Z.Y.; Feng, Z.Q.; Feng, L.; Liao, L.S. Secondary Grain Growth of Perovskite Films Induced by a Phosphonate Derivative for High-Performance Near-Infrared Light–Emitting Diodes. *Advanced Functional Materials* 2025, *35*, 2504744.
2. Wei, J.; Li, J.; Duan, C.; Yuan, L.; Zou, S.; Pang, Q.; Yan, K. High Efficiency Near-Infrared Perovskite Light Emitting Diodes With Reduced Rolling-Off by Surface Post-Treatment. *Small* 2023, *19*, 2207769.
3. Deng, Y.; Zhang, Z.; Ren, G.; Li, Z.; Liu, C.; Guo, W. An easily developed difunctional molecule enabling highly efficient and stable light-emitting diodes. *Advanced Functional Materials* 2023, *33*, 2305423.
4. Du, J.; Huang, Y.; Yun, Y.; Wei, W.; Jiang, S.; Yang, Y.; Chen, M.; Li, C. Interface permanent dipoles induced by atomic layer deposited Al2O3 for high-performance perovskite light-emitting diodes. *Applied Surface Science* 2025, *700*, 163205.
5. Zhao, X.; Tan, Z.-K. Large-area near-infrared perovskite light-emitting diodes. *Nature Photonics* 2020, *14*, 215–218.
6. Liu, H.; Shi, G.; Khan, R.; Chu, S.; Huang, Z.; Shi, T.; Sun, H.; Li, Y.; Zhou, H.; Xiao, P. Large-area flexible perovskite light-emitting diodes enabled by inkjet printing. *Advanced Materials* 2024, *36*, 2309921.
7. Gao, X.-Y.; Wu, X.-Y.; Liu, W.-Z.; Xu, S.-H.; Zhou, D.-Y.; Liao, L.-S. Synergistic Interface–Bulk Passivation Induced by a Pre-Additive Modifier for Efficient Near-Infrared Perovskite Light-Emitting Diodes. *ACS Applied Energy Materials* 2026, *9*, 3350–3359.
8. Din, N.; Saeed, F.; Hussain, S.; Sultan, R.M.D.; Keles, S.; Salman, M.; Naqvi, S.M.Q.R.; Belaid, I.; Khan, Q.; Lei, W. Fabrication of stable perovskite-based near-infrared light emitting devices for noninvasive bioimaging applications. *Materials Today Advances* 2026, *30*, 100775.
9. Li, M.; Yang, Y.; Kuang, Z.; Hao, C.; Wang, S.; Lu, F.; Liu, Z.; Liu, J.; Zeng, L.; Cai, Y. Acceleration of radiative recombination for efficient perovskite LEDs. *Nature* 2024, *630*, 631–635.

Table S2. TRPL fitting parameters of perovskite film without and with PEDOT:PSS

Perovskite film	τ_1 (ns)	τ_2 (ns)	A1	A2	τ_{ave} (ns)
Without the PEDOT:PSS buffer layer	38.82	280.90	0.66	0.25	217.04
With the PEDOT:PSS buffer layer	49.12	449.52	0.66	0.23	353.51
